# 2,5-Bis(pyridinium-2-yl)-3,6-bis­(2-pyrid­yl)pyrazine bis­[tetra­chlorido­aurate(III)]

**DOI:** 10.1107/S1600536811036208

**Published:** 2011-09-14

**Authors:** Anita Abedi, Azra Dabbaghi, Vahid Amani

**Affiliations:** a Department of Chemistry, North Tehran Branch, Islamic Azad University, Tehran, Iran; b Department of Chemistry, Share-Ray Branch, Islamic Azad University, Tehran, Iran

## Abstract

In the title compound, (C_24_H_18_N_6_)[AuCl_4_]_2_, the cation is located on an inversion center. Each of the two independent Au^III^ ions lies on an inversion center and has a distorted square-planar geometry. In the crystal, inter­molecular C—H⋯Cl hydrogen bonds, π–π inter­actions [centroid–centroid distances = 3.5548 (16) and 3.7507 (16) Å] and Au⋯π inter­actions [Au⋯centroid distance = 3.6424 (10) Å] are effective in the stabilization of the structure, resulting in the formation of a supra­molecular structure. Intra­molecular N—H⋯N hydrogen bonds are present in the cation.

## Related literature

For the structures of related proton-transfer complexes, see: Abedi *et al.* (2008 ▶); Aragoni *et al.* (2005*a* ▶,*b* ▶); Bock *et al.* (1992 ▶); Calleja *et al.* (2001 ▶); Graf & Stoeckli-Evans (1996 ▶); Hasan *et al.* (1999 ▶); Hojjat Kashani *et al.* (2008 ▶); Johnson & Steed (1998 ▶); Kalateh *et al.* (2008 ▶); Padgett *et al.* (2005 ▶); Yap *et al.* (1995 ▶); Yıldırım *et al.* (2009*a* ▶,*b* ▶); Zhang *et al.* (2006 ▶).
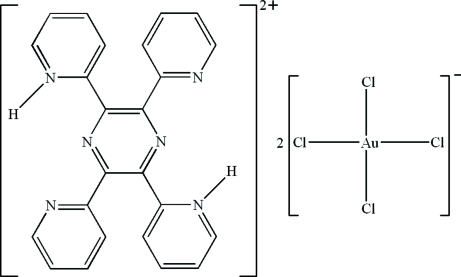


## Experimental

### 

#### Crystal data

(C_24_H_18_N_6_)[AuCl_4_]_2_
*M* *_r_* = 1067.98
Triclinic, 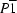

*a* = 7.2847 (6) Å
*b* = 9.6611 (8) Å
*c* = 10.6263 (9) Å
α = 79.6692 (13)°
β = 78.7378 (12)°
γ = 88.8600 (13)°
*V* = 721.48 (10) Å^3^
*Z* = 1
Mo *K*α radiation
μ = 10.93 mm^−1^
*T* = 100 K
0.30 × 0.20 × 0.20 mm

#### Data collection

Bruker APEXII CCD diffractometer
Absorption correction: multi-scan (*SADABS*; Sheldrick, 1996 ▶) *T*_min_ = 0.080, *T*_max_ = 0.110
8652 measured reflections
3821 independent reflections
3395 reflections with *I* > 2σ(*I*)
*R*_int_ = 0.020

#### Refinement

*R*[*F* ^2^ > 2σ(*F* ^2^)] = 0.019
*wR*(*F* ^2^) = 0.050
*S* = 1.05
3821 reflections
185 parameters
H-atom parameters constrained
Δρ_max_ = 1.76 e Å^−3^
Δρ_min_ = −1.36 e Å^−3^

### 

Data collection: *APEX2* (Bruker, 2007 ▶); cell refinement: *SAINT* (Bruker, 2007 ▶); data reduction: *SAINT*; program(s) used to solve structure: *SHELXS97* (Sheldrick, 2008 ▶); program(s) used to refine structure: *SHELXL97* (Sheldrick, 2008 ▶); molecular graphics: *SHELXTL* (Sheldrick, 2008 ▶) and *Mercury* (Macrae *et al.*, 2006 ▶); software used to prepare material for publication: *SHELXTL*.

## Supplementary Material

Crystal structure: contains datablock(s) I. DOI: 10.1107/S1600536811036208/hy2466sup1.cif

Structure factors: contains datablock(s) I. DOI: 10.1107/S1600536811036208/hy2466Isup2.hkl

Additional supplementary materials:  crystallographic information; 3D view; checkCIF report

## Figures and Tables

**Table 1:** Selected bond lengths (Å)

Au1—Cl1	2.2775 (7)
Au1—Cl2	2.2774 (6)
Au2—Cl3	2.2834 (7)
Au2—Cl4	2.2821 (6)

**Table 2:** Hydrogen-bond geometry (Å, °)

*D*—H⋯*A*	*D*—H	H⋯*A*	*D*⋯*A*	*D*—H⋯*A*
N2—H2*N*⋯N3^i^	0.87	1.69	2.538 (3)	164
C4—H4*A*⋯Cl1^ii^	0.95	2.77	3.680 (3)	161
C6—H6*A*⋯Cl2^iii^	0.95	2.77	3.519 (3)	136

Symmetry codes: (i) 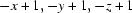; (ii) 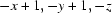; (iii) 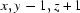.

**Fig. 1.**
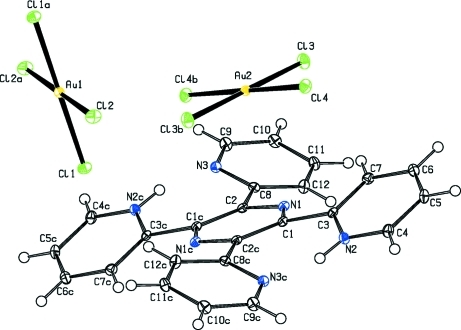
The molecular structure of the title compound. Displacement ellipsoids are drawn at the 50% probability level. [Symmetry codes: (a) -x, 2-y, -z; (b) -x, 1-y, 1-z; (c) 1-x, 1-y, 1-z.]

**Fig. 2.**
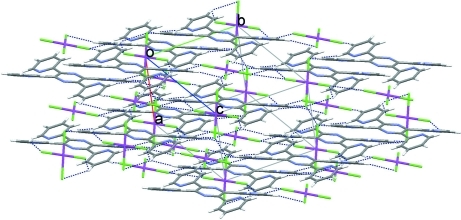
Crystal packing diagram for the title compound. Hydrogen bonds are shown as dashed lines.

## supplementary crystallographic information

### Comment

Recently, we reported the synthesis and crystal structures of two proton-transfer complexes (Abedi *et al.*, 2008; Kalateh *et al.*, 2008). Several proton-transfer systems using 2,3,5,6-tetrakis(2-pyridyl)pyrazine (tppz) as proton donor molecules, such as [tppzH_2_][I_3_]_2_.2I_2_, (II), [tppzH_4_][I_3_]_2_[I]_2_, (III), [tppzH_4_][Br]_4_.2H_2_O, (IV), (Padgett *et al.*, 2005), [tppzH_4_][Br]_2_[Br_4_], (V), (Aragoni *et al.*, 2005*a*), [tppzH_2_][ICl_2_]_2_, (VI), (Aragoni *et al.*, 2005*b*), [tppzH_4_][Cl]_4_.2H_2_O, (VII), (Graf & Stoeckli-Evans, 1996) and [tppzH_2_][B(Ph)_4_]_2_, (IIX), (Bock *et al.*, 1992), have been synthesized and characterized by single-crystal X-ray diffraction methods. Several proton-transfer systems using AuCl_4_ as proton acceptor molecules, such as [EMI][AuCl_4_], (IX), [BMI]_2_[AuCl_4_].2H_2_O, (X), (Hasan *et al.*, 1999), [H_2_bipy][AuCl_4_][Cl], (XI), (Zhang *et al.*, 2006), [H_7_O_3_][15-crown-5][AuCl_4_], (XII), [H_5_O_2_][benzo-15-crown-5]_2_[AuCl_4_], (XIII), (Johnson & Steed, 1998), [H_5_O_2_]_2_[12-crown-4]_2_[AuCl_4_]_2_, (XIV), [H_3_O][18-crown-6][AuCl_4_], (XV), [H_3_O][4-nitrobenzo-18-crown-6][AuCl_4_], (XVI), (Calleja *et al.*, 2001), [DPpyH][AuCl_4_], (XVII), (Yap *et al.*, 1995), [H_2_DA18C6][AuCl_4_], (XVIII), (Hojjat Kashani *et al.*, 2008), [Me_2_Ph_2_phenH][AuCl_4_], (XIX), (Yıldırım *et al.*, 2009*a*) and [pz(py)_2_H][AuCl_4_], (XX), (Yıldırım *et al.*, 2009*b*) (EMI is 1-ethyl-3-methylimidazolium, BMI is 1-butyl-3-methylimidazolium, H_2_bipy is 2,2'-bipyridinium, DPpyH is 2,6-diphenylpyridinium, H_2_DA18C6 is 1,10-diazonia-18-crown-6, Me_2_Ph_2_phenH is 2,9-dimethyl-4,7-diphenyl-1,10-phenanthrolin-1-ium and pz(py)_2_H is 2-[3-(2-pyridyl)pyrazin-2-yl]pyridinium) have been synthesized and characterized by single-crystal X-ray diffraction methods. We report herein the synthesis and crystal structure of the title compound, (I).

The asymmetric unit of (I) contains one half-cation and two half-anions (Fig. 1). The Au^III^ ions, each lies on an inversion center, have a square-planer coordination geometry. The bond lengths and angles in the cation are in good agreement with the corresponding values in (V) and (IIX). The Au—Cl bond lengths (Table 1) and angles are within normal range observed in (XIIX), (XIX) and (XX). In the crystal structure, intermolecular C—H···Cl hydrogen bonds (Table 2), π–π contacts between the pyridine rings (Fig. 2), *Cg*2···*Cg*2^i^ and *Cg*3···*Cg*3^ii^ [*Cg*2 and *Cg*3 are the centroids of the N2, C3–C7 ring and N3, C8–C12 ring. Symmetry codes: (i) 1-*x*, -y, 1-*z*; (ii) 1-*x*, 1-*y*, 2-*z*], with centroid–centroid distances of 3.5548 (16) and 3.7507 (16) Å and Au2···*Cg*1 contacts (Fig. 2) [*Cg*1 is the centroid of the N1, C1, C2, N1^iii^, C1^iii^, C2^iii^ ring. Symmetry code: (iii) 1-*x*, 1-*y*, 1-*z*] with an Au···centroid distance of 3.6424 (10) Å are effective in the stabilization of the crystal structure, resulting in the formation of a supramolecular structure.

### Experimental

For the preparation of (I), a solution of 2,3,5,6-tetrakis(2-pyridyl)pyrazine (0.26 g, 0.65 mmol) in CHCl_3_ (20 ml) was added to a solution of HAuCl_4_.3H_2_O, (0.45 g, 1.30 mmol) in methanol (20 ml) and the resulting yellow solution was stirred for 10 min at room temperature. Crystals suitable for X-ray diffraction experiment were obtained by methanol diffusion into a yellow solution in DMF. After one week, yellow prismatic crystals of (I) were isolated (yield: 0.51 g, 73.5%; m. p. 569–570 K).

### Refinement

H atoms were positioned geometrically and refined as riding atoms, with C—H = 0.95 and N—H = 0.87 Å and with *U*_iso_(H) = 1.2*U*_eq_(C,N). The highest residual electron density was found at 0.82 Å from Au2 atom and the deepest hole at 0.76 Å from Au1 atom.

### Crystal data

**Table:**

(C_24_H_18_N_6_)[AuCl_4_]_2_	*Z* = 1
*M**_r_* = 1067.98	*F*(000) = 498
Triclinic, *P*1	*D*_x_ = 2.458 Mg m^−^^3^
Hall symbol: -P 1	Mo *K*α radiation, λ = 0.71073 Å
*a* = 7.2847 (6) Å	Cell parameters from 336 reflections
*b* = 9.6611 (8) Å	θ = 3.0–29.0°
*c* = 10.6263 (9) Å	µ = 10.93 mm^−^^1^
α = 79.6692 (13)°	*T* = 100 K
β = 78.7378 (12)°	Prism, yellow
γ = 88.8600 (13)°	0.30 × 0.20 × 0.20 mm
*V* = 721.48 (10) Å^3^	

### Data collection

**Table:**

Bruker APEXII CCD diffractometer	3821 independent reflections
Radiation source: fine-focus sealed tube	3395 reflections with *I* > 2σ(*I*)
graphite	*R*_int_ = 0.020
φ and ω scans	θ_max_ = 29.0°, θ_min_ = 2.0°
Absorption correction: multi-scan (*SADABS*; Sheldrick, 1996)	*h* = −9→9
*T*_min_ = 0.080, *T*_max_ = 0.110	*k* = −13→13
8652 measured reflections	*l* = −14→14

### Refinement

**Table:**

Refinement on *F*^2^	Secondary atom site location: difference Fourier map
Least-squares matrix: full	Hydrogen site location: inferred from neighbouring sites
*R*[*F*^2^ > 2σ(*F*^2^)] = 0.019	H-atom parameters constrained
*wR*(*F*^2^) = 0.050	*w* = 1/[σ^2^(*F*_o_^2^) + (0.0278*P*)^2^ + 0.1485*P*] where *P* = (*F*_o_^2^ + 2*F*_c_^2^)/3
*S* = 1.05	(Δ/σ)_max_ = 0.003
3821 reflections	Δρ_max_ = 1.76 e Å^−^^3^
185 parameters	Δρ_min_ = −1.36 e Å^−^^3^
0 restraints	Extinction correction: *SHELXL*, Fc^*^=kFc[1+0.001xFc^2^λ^3^/sin(2θ)]^-1/4^
Primary atom site location: structure-invariant direct methods	Extinction coefficient: 0.0137 (4)

### Fractional atomic coordinates and isotropic or equivalent isotropic displacement parameters (Å^2^)

**Table:**

	*x*	*y*	*z*	*U*_iso_*/*U*_eq_	
N1	0.4305 (3)	0.3824 (2)	0.5837 (2)	0.0090 (4)	
N2	0.4962 (3)	0.2025 (2)	0.3140 (2)	0.0103 (4)	
H2N	0.5714	0.2659	0.2616	0.012*	
N3	0.3379 (3)	0.5836 (2)	0.8362 (2)	0.0102 (4)	
C1	0.4712 (3)	0.3792 (3)	0.4559 (2)	0.0095 (5)	
C2	0.4559 (3)	0.4976 (3)	0.6317 (2)	0.0091 (5)	
C3	0.4186 (3)	0.2409 (3)	0.4284 (2)	0.0084 (5)	
C4	0.4503 (4)	0.0810 (3)	0.2834 (3)	0.0125 (5)	
H4A	0.5062	0.0580	0.2014	0.015*	
C5	0.3216 (4)	−0.0119 (3)	0.3702 (3)	0.0126 (5)	
H5A	0.2882	−0.0979	0.3483	0.015*	
C6	0.2435 (4)	0.0241 (3)	0.4889 (3)	0.0130 (5)	
H6A	0.1563	−0.0380	0.5503	0.016*	
C7	0.2919 (4)	0.1504 (3)	0.5190 (3)	0.0117 (5)	
H6B	0.2388	0.1749	0.6009	0.014*	
C8	0.3936 (3)	0.4731 (3)	0.7766 (2)	0.0092 (5)	
C9	0.2659 (4)	0.5627 (3)	0.9642 (3)	0.0136 (5)	
H8A	0.2255	0.6416	1.0038	0.016*	
C10	0.2484 (4)	0.4302 (3)	1.0404 (3)	0.0135 (5)	
H9A	0.1943	0.4174	1.1307	0.016*	
C11	0.3120 (4)	0.3162 (3)	0.9817 (3)	0.0126 (5)	
H10A	0.3062	0.2242	1.0323	0.015*	
C12	0.3843 (4)	0.3378 (3)	0.8486 (2)	0.0127 (5)	
H11A	0.4269	0.2605	0.8072	0.015*	
Cl3	−0.10327 (10)	0.34900 (7)	0.69144 (6)	0.01701 (14)	
Cl4	−0.05043 (9)	0.33137 (7)	0.38464 (6)	0.01462 (13)	
Cl1	0.28009 (9)	0.92195 (7)	0.04725 (6)	0.01430 (13)	
Cl2	0.05623 (9)	0.89691 (7)	−0.18017 (6)	0.01601 (14)	
Au1	0.0000	1.0000	0.0000	0.00864 (5)	
Au2	0.0000	0.5000	0.5000	0.00909 (5)	

### Atomic displacement parameters (Å^2^)

**Table:**

	*U*^11^	*U*^22^	*U*^33^	*U*^12^	*U*^13^	*U*^23^
N1	0.0094 (10)	0.0098 (10)	0.0087 (10)	0.0017 (8)	−0.0029 (8)	−0.0026 (8)
N2	0.0119 (10)	0.0098 (10)	0.0087 (10)	−0.0012 (8)	−0.0001 (8)	−0.0018 (8)
N3	0.0111 (10)	0.0103 (10)	0.0089 (10)	0.0001 (8)	−0.0009 (8)	−0.0023 (8)
C1	0.0094 (11)	0.0085 (11)	0.0107 (11)	0.0018 (9)	−0.0009 (9)	−0.0031 (9)
C2	0.0081 (11)	0.0096 (11)	0.0098 (11)	0.0019 (9)	−0.0014 (9)	−0.0032 (9)
C3	0.0090 (11)	0.0083 (11)	0.0086 (11)	0.0025 (9)	−0.0034 (9)	−0.0019 (9)
C4	0.0162 (13)	0.0105 (12)	0.0113 (12)	0.0011 (10)	−0.0021 (10)	−0.0043 (10)
C5	0.0172 (13)	0.0078 (11)	0.0136 (12)	−0.0004 (10)	−0.0046 (10)	−0.0021 (10)
C6	0.0140 (13)	0.0108 (12)	0.0139 (13)	−0.0018 (10)	−0.0037 (10)	−0.0004 (10)
C7	0.0125 (12)	0.0122 (12)	0.0098 (12)	0.0016 (10)	−0.0004 (10)	−0.0029 (10)
C8	0.0089 (11)	0.0113 (12)	0.0072 (11)	−0.0009 (9)	−0.0004 (9)	−0.0027 (9)
C9	0.0172 (13)	0.0136 (13)	0.0101 (12)	−0.0016 (10)	−0.0001 (10)	−0.0053 (10)
C10	0.0161 (13)	0.0160 (13)	0.0079 (11)	−0.0018 (10)	−0.0005 (10)	−0.0024 (10)
C11	0.0160 (13)	0.0112 (12)	0.0103 (12)	−0.0026 (10)	−0.0023 (10)	−0.0007 (10)
C12	0.0177 (13)	0.0111 (12)	0.0091 (12)	0.0010 (10)	−0.0014 (10)	−0.0024 (9)
Cl3	0.0223 (3)	0.0147 (3)	0.0120 (3)	−0.0025 (3)	0.0002 (3)	−0.0005 (2)
Cl4	0.0180 (3)	0.0130 (3)	0.0151 (3)	0.0005 (2)	−0.0051 (3)	−0.0063 (2)
Cl1	0.0133 (3)	0.0173 (3)	0.0133 (3)	0.0026 (2)	−0.0028 (2)	−0.0054 (2)
Cl2	0.0173 (3)	0.0215 (3)	0.0111 (3)	0.0019 (3)	−0.0008 (2)	−0.0103 (2)
Au1	0.01007 (8)	0.00904 (8)	0.00661 (8)	−0.00065 (5)	0.00033 (5)	−0.00288 (5)
Au2	0.00902 (8)	0.00935 (8)	0.00916 (8)	0.00064 (5)	−0.00137 (5)	−0.00284 (5)

### Geometric parameters (Å, °)

**Table:**

N1—C2	1.335 (3)	C6—C7	1.384 (4)
N1—C1	1.338 (3)	C6—H6A	0.9500
N2—C4	1.338 (3)	C7—H6B	0.9500
N2—C3	1.349 (3)	C8—C12	1.387 (4)
N2—H2N	0.8705	C9—C10	1.380 (4)
N3—C9	1.339 (3)	C9—H8A	0.9500
N3—C8	1.353 (3)	C10—C11	1.389 (4)
C1—C2^i^	1.414 (4)	C10—H9A	0.9500
C1—C3	1.491 (3)	C11—C12	1.389 (3)
C2—C1^i^	1.414 (4)	C11—H10A	0.9500
C2—C8	1.494 (3)	C12—H11A	0.9500
C3—C7	1.388 (3)	Au1—Cl1	2.2775 (7)
C4—C5	1.391 (4)	Au1—Cl2	2.2774 (6)
C4—H4A	0.9500	Au2—Cl3	2.2834 (7)
C5—C6	1.381 (4)	Au2—Cl4	2.2821 (6)
C5—H5A	0.9500		
			
C2—N1—C1	122.5 (2)	C3—C7—H6B	120.3
C4—N2—C3	121.8 (2)	N3—C8—C12	120.3 (2)
C4—N2—H2N	124.3	N3—C8—C2	119.3 (2)
C3—N2—H2N	113.8	C12—C8—C2	120.4 (2)
C9—N3—C8	120.3 (2)	N3—C9—C10	122.1 (3)
N1—C1—C2^i^	118.8 (2)	N3—C9—H8A	118.9
N1—C1—C3	111.4 (2)	C10—C9—H8A	118.9
C2^i^—C1—C3	129.6 (2)	C9—C10—C11	118.3 (2)
N1—C2—C1^i^	118.7 (2)	C9—C10—H9A	120.9
N1—C2—C8	111.2 (2)	C11—C10—H9A	120.9
C1^i^—C2—C8	130.0 (2)	C12—C11—C10	119.5 (2)
N2—C3—C7	119.4 (2)	C12—C11—H10A	120.2
N2—C3—C1	119.6 (2)	C10—C11—H10A	120.2
C7—C3—C1	121.0 (2)	C8—C12—C11	119.4 (2)
N2—C4—C5	120.8 (2)	C8—C12—H11A	120.3
N2—C4—H4A	119.6	C11—C12—H11A	120.3
C5—C4—H4A	119.6	Cl2^ii^—Au1—Cl2	180.0
C6—C5—C4	118.2 (2)	Cl2^ii^—Au1—Cl1	90.28 (2)
C6—C5—H5A	120.9	Cl2—Au1—Cl1	89.72 (2)
C4—C5—H5A	120.9	Cl1—Au1—Cl1^ii^	180.0
C5—C6—C7	120.3 (3)	Cl4^iii^—Au2—Cl4	180.000 (19)
C5—C6—H6A	119.9	Cl4^iii^—Au2—Cl3	89.50 (2)
C7—C6—H6A	119.9	Cl4—Au2—Cl3	90.50 (2)
C6—C7—C3	119.4 (2)	Cl3—Au2—Cl3^iii^	180.0
C6—C7—H6B	120.3		
			
C2—N1—C1—C2^i^	0.1 (4)	N2—C3—C7—C6	−1.8 (4)
C2—N1—C1—C3	−176.5 (2)	C1—C3—C7—C6	178.7 (2)
C1—N1—C2—C1^i^	−0.1 (4)	C9—N3—C8—C12	−2.9 (4)
C1—N1—C2—C8	178.2 (2)	C9—N3—C8—C2	174.0 (2)
C4—N2—C3—C7	2.1 (4)	N1—C2—C8—N3	−153.8 (2)
C4—N2—C3—C1	−178.4 (2)	C1^i^—C2—C8—N3	24.3 (4)
N1—C1—C3—N2	−160.6 (2)	N1—C2—C8—C12	23.1 (3)
C2^i^—C1—C3—N2	23.3 (4)	C1^i^—C2—C8—C12	−158.8 (3)
N1—C1—C3—C7	19.0 (3)	C8—N3—C9—C10	1.2 (4)
C2^i^—C1—C3—C7	−157.2 (3)	N3—C9—C10—C11	1.5 (4)
C3—N2—C4—C5	−1.0 (4)	C9—C10—C11—C12	−2.4 (4)
N2—C4—C5—C6	−0.4 (4)	N3—C8—C12—C11	1.9 (4)
C4—C5—C6—C7	0.7 (4)	C2—C8—C12—C11	−174.9 (2)
C5—C6—C7—C3	0.4 (4)	C10—C11—C12—C8	0.7 (4)

Symmetry codes: (i) −*x*+1, −*y*+1, −*z*+1; (ii) −*x*, −*y*+2, −*z*; (iii) −*x*, −*y*+1, −*z*+1.

### Hydrogen-bond geometry (Å, °)

**Table:**

*D*—H···*A*	*D*—H	H···*A*	*D*···*A*	*D*—H···*A*
N2—H2N···N3^i^	0.87	1.69	2.538 (3)	164
C4—H4A···Cl1^iv^	0.95	2.77	3.680 (3)	161
C6—H6A···Cl2^v^	0.95	2.77	3.519 (3)	136

Symmetry codes: (i) −*x*+1, −*y*+1, −*z*+1; (iv) −*x*+1, −*y*+1, −*z*; (v) *x*, *y*−1, *z*+1.

## References

[bb1] Abedi, A., Bahrami Shabestari, A. & Amani, V. (2008). *Acta Cryst.* E**64**, o990. 10.1107/S1600536808012579 PMC2961481 21202715

[bb2] Aragoni, M. C., Arca, M., Devillanova, F. A., Hursthouse, M. B., Huth, S. L., Isaia, F., Lippolis, V., Mancini, A. & Ogilvie, H. (2005*a*). *Inorg. Chem. Commun.* **8**, 79–82.

[bb3] Aragoni, M. C., Arca, M., Devillanova, F. A., Hursthouse, M. B., Huth, S. L., Isaia, F., Lippolis, V., Mancini, A., Ogilvie, H. & Verani, G. (2005*b*). *J. Organomet. Chem.* **690**, 1923–1934.

[bb4] Bock, H., Vaupel, T., Näther, C., Ruppert, K. & Havlas, Z. (1992). *Angew. Chem. Int. Ed.* **31**, 299–301.

[bb5] Bruker (2007). *APEX2* and *SAINT* Bruker AXS Inc., Madison, Wisconsin, USA.

[bb6] Calleja, M., Johnson, K., Belcher, W. J. & Steed, W. (2001). *Inorg. Chem.* **40**, 4978–4985. 10.1021/ic010468i 11531447

[bb7] Graf, M. & Stoeckli-Evans, H. (1996). *Acta Cryst.* C**52**, 3073–3075.

[bb8] Hasan, M., Kozhevnikov, I. V., Siddiqu, M. R. H., Steiner, A. & Winterton, N. (1999). *Inorg. Chem.* **38**, 5637–5641. 10.1021/ic000606o 11225125

[bb9] Hojjat Kashani, L., Yousefi, M., Amani, V. & Khavasi, H. R. (2008). *Acta Cryst.* E**64**, m840–m841. 10.1107/S1600536808015353 PMC2961538 21202520

[bb10] Johnson, K. & Steed, J. W. (1998). *Chem. Commun.* pp. 1479–1480.

[bb11] Kalateh, K., Ebadi, A., Abedi, A., Amani, V. & Khavasi, H. R. (2008). *Acta Cryst.* E**64**, m1267–m1268. 10.1107/S160053680802881X PMC2959317 21201020

[bb12] Macrae, C. F., Edgington, P. R., McCabe, P., Pidcock, E., Shields, G. P., Taylor, R., Towler, M. & van de Streek, J. (2006). *J. Appl. Cryst.* **39**, 453–457.

[bb13] Padgett, C. W., Walsh, R. D., Drake, G. W., Hanks, T. W. & Pennington, W. T. (2005). *Cryst. Growth Des.* **5**, 745–753.

[bb14] Sheldrick, G. M. (1996). *SADABS* University of Göttingen, Germany.

[bb15] Sheldrick, G. M. (2008). *Acta Cryst.* A**64**, 112–122. 10.1107/S0108767307043930 18156677

[bb16] Yap, G. P. A., Rheingold, A. R., Das, P. & Crabtree, R. H. (1995). *Inorg. Chem.* **34**, 3474–3476.

[bb17] Yıldırım, S. Ö., Akkurt, M., Safari, N., Abedi, A., Amani, V. & McKee, V. (2009*a*). *Acta Cryst.* E**65**, m479–m480. 10.1107/S1600536809011994 PMC2977548 21583734

[bb18] Yıldırım, S. Ö., Akkurt, M., Safari, N., Amani, V. & McKee, V. (2009*b*). *Acta Cryst.* E**65**, m491–m492. 10.1107/S1600536809012264 PMC2977556 21583742

[bb19] Zhang, X.-P., Yang, G. & Ng, S. W. (2006). *Acta Cryst.* E**62**, m2018–m2020.

